# Effect of Random Lateral Ballast Resistance on Force-Deformation Characteristics of CWR with a Small-Radius Curve

**DOI:** 10.3390/ma16072876

**Published:** 2023-04-04

**Authors:** Huan Wang, Chengwei Xing, Xiaohui Deng

**Affiliations:** 1 School of Highway, Chang’an University, Xi’an 710064, China; xingcw@chd.edu.cn; 2 China Railway Fifth Survey and Design Institute Group Co., Ltd., Beijing 102600, China

**Keywords:** Shapiro–Wilk test, lateral ballast resistance, finite element model, continuous welded rail

## Abstract

To address the randomness of lateral ballast resistance in the field and its effect on the force-deformation behavior of Continuous Welded Rail (CWR) with small-radius curves, field tests were first conducted to investigate longitudinal and lateral ballast resistance on a 250 m-radius curve. It was found that the lateral ballast resistance follows a normal distribution based on the Shapiro–Wilk test. A finite element model of a small-radius curve CWR track was then established based on actual field conditions, and the force-deformation characteristics were analyzed under thermal loading. The results showed that it is of great significance to incorporate the randomness of the lateral ballast resistance as the deformation mode is closer to the actual field situation. In particular, attention should be given to areas where the lateral ballast resistance is weak. The research presented here has significant implications for railway maintenance practice.

## 1. Introduction

Transportation infrastructures represented by railways and roads contribute greatly to economic development. The characteristics of granular material including ballast and aggregates, concrete, asphalt, steel, and so on, as well as their interactions, directly affect the mechanical properties and service performance of transportation infrastructure [1,2,3]. The problems related to the use of seamed tracks especially on small-radius curves have become progressively more serious, comprising joint deterioration, train swaying, elevated maintenance expenses, and jeopardized running safety. As a result, continuous welded rail (CWR) has been replacing seamed tracks over the past few decades and continues to do so today due to its superior performance, which includes reduced maintenance costs, extended service life, and improved passenger comfort. CWR tracks, especially for small-radius curves, however, have certain drawbacks including buckling and broken failure. Therefore, its use is regulated by various railroad administrations and international organizations. For instance, before 2014, the National Railway Administration of China did not permit the laying of CWR tracks on curves with a radius smaller than 300 m. Therefore, a comprehensive study was conducted in Hebei province to investigate the CWR laying of 250 m-radius curves for understanding and controlling the drawbacks [4]. A potential drawback of CWR is track buckling in the lateral plane, which is the most significant issue that needs to be understood and controlled [5]. Aside from significant deflection buckling, CWR tracks can also experience issues such as lateral shifting and radial breathing [6]. In summer, compressive loads in the rails cause the track to move outward; the compressive forces caused by thermal loads can be described based on the following relation [7]:

(1) N=EAαΔT

where N is a compressive force;

E denotes elastic modulus;

A denotes a cross-sectional area;

α denotes the thermal expansion coefficient;

ΔT denotes the temperature difference between rail and neutral temperature.

Compared with a tangent section or curved section with a larger radius, the buckling issue, lateral shifting, and radial breathing are more severe in smaller radius curves since the radial thermal force and deformation in CWR increase as the curvature radius decreases [8]. This has negative consequences for the track as it reduces the lateral resistance and can create localized lateral misalignments, even leading to buckling. Particularly, when there is weak lateral restraint and high thermal forces, this movement is rarely nonuniform [9], which is the main source of rail irregularity that may affect passenger comfort [10,11,12,13] and vehicle-overhead infrastructure interaction [14]. In the worst-case scenario, it could even pose a safety risk, e.g., a vehicle derailment [15]. Hence, it is of great significance to monitor displacement for CWR tracks of small-radius curves [16].

To maintain the stability and alignment of CWR, lateral ballast resistance is considered to be the most important factor [17] and contributes 60% and even up to 70% to the lateral strength [18,19]. Other influencing factors include track curvature, initial misalignment, and longitudinal and torsional stiffness. To measure the lateral ballast resistance, Single Tie Push Test (STPT) and Lateral Track Panel Loading Test (LTPT) [20,21] are generally adopted, and various factors are found to affect lateral ballast resistance, including ballast consolidation [22], sleeper type [23,24,25], maintenance practice [19,26], etc. These factors mentioned may exhibit variations along the track and lead to the variability of the lateral ballast resistance [27,28,29,30]. However, existing studies have not adequately addressed the overall randomness of lateral ballast resistance in the field or its effect on the actual force-deformation behavior of the CWR track.

Although this 250-m-radius-curve CWR has been successfully laid in Hebei province, certain technical challenges persist in terms of daily maintenance. Empirical knowledge about the characterization of lateral ballast resistance randomness is not clear. Moreover, the uncertainty introduced by the randomness to the real force-deformation behavior of CWR with this small-radius curve is still lacking.

In this article, field tests are firstly conducted on a 250-m radius curve, followed by characterization of the randomness of lateral ballast resistance through Shapiro–Wilk (SW) testing. Subsequently, a numerical study is performed to examine the force-deformation characteristics, which serves as a crucial foundation and initial step for further buckling analysis. The nomenclature employed in this study can be found in Table A1.

## 2. Field Tests

The test was conducted at a curved section of the CWR track with a radius of 250 m and a design speed of 120 km/h. The region has a maximum rail temperature difference of 84.8 °C. A total of 13 sleepers were randomly sampled for longitudinal ballast resistance testing, and 15 sleepers were randomly sampled for lateral ballast resistance testing. The distance between random samples was maintained at 6–10 sleeper spacing to prevent any possible disturbances. The track under test is composed of standard Type III concrete sleepers and standard 60 kg/m rail in China [31]. The spacing of the sleeper is 0.6 m, and the track gauge is 1.435 m. The ballast layer in normal conditions consists of first-class granite and has a shoulder width of 500 mm and a height of 150 mm, with a slope of 1:1.75. Longitudinal ballast resistance is crucial to resisting longitudinal forces generated by thermal gradient, dynamic braking, and rail creep offered by sleepers and ballast to the rails. As previously stated, the lateral deformation characteristics of CWR are significantly influenced by lateral ballast resistance, which is considered the most important factor in maintaining CWR alignment and stability. For random sampling purposes, a widely-used method STPT is utilized to characterize the force-displacement relationship, in which the displacement is determined by measuring sleeper displacement, and the force is determined by measuring the pushing force.

### 2.1. Testing Method

**Figure 1:**
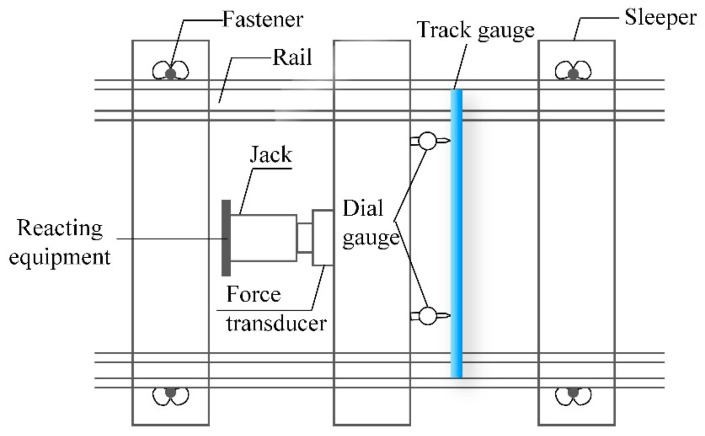
Test schematic diagram of longitudinal ballast resistance.

**Figure 2:**
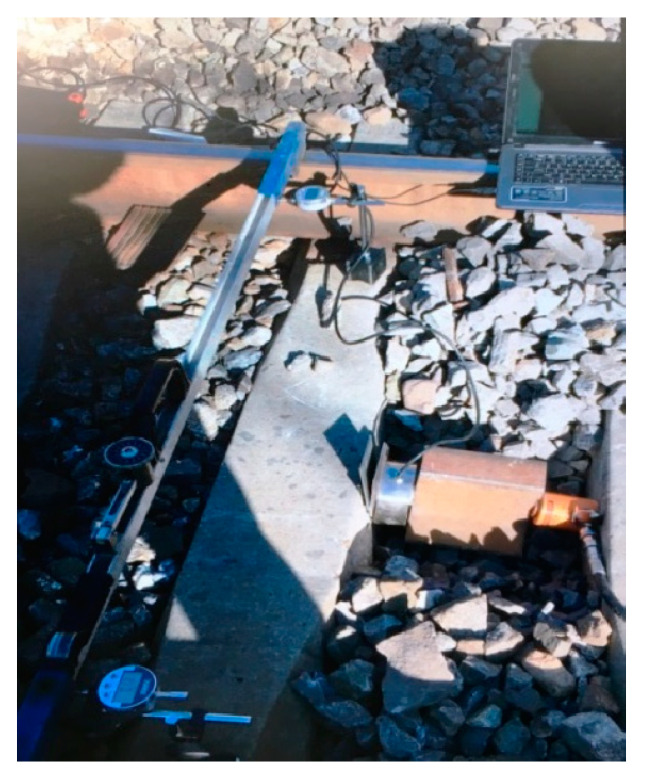
Field tests of longitudinal ballast resistance.

To determine longitudinal resistance, the fasteners on the measured sleeper were loosened, and the rail pad was removed. A hydraulic jack was used to apply longitudinal thrust, and its value was recorded by a digital instrument display. A pressure sensor was placed between the hydraulic jack and the digital instrument display while reacting equipment provides the longitudinal reaction force. The location for installing the reacting equipment was determined based on the range of the hydraulic jack and the thickness of the pressure sensor. The displacement of the rail sleeper was recorded using a dial gauge. The test schematic and field test diagrams are presented in Figure 1 and Figure 2, respectively.

**Figure 3:**
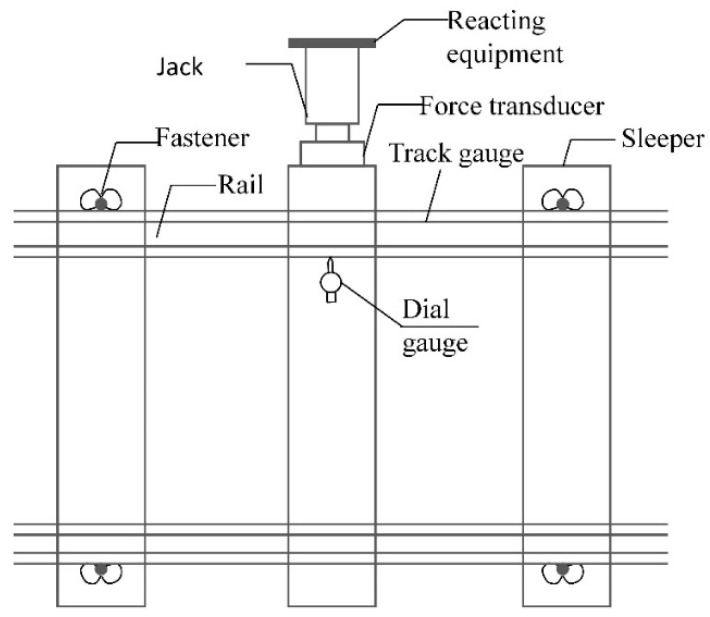
Test schematic diagram of lateral ballast resistance.

**Figure 4:**
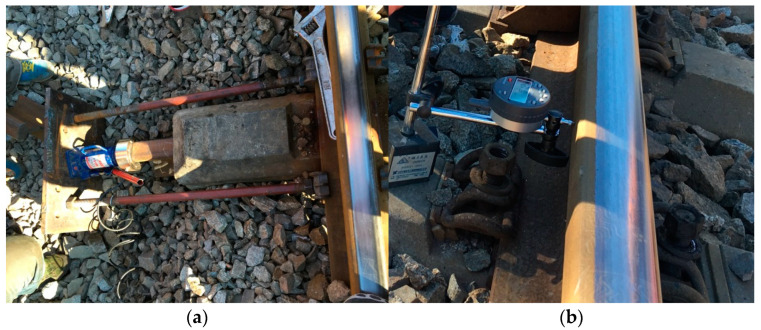
Field tests of lateral ballast resistance. (**a**) Forcing device; (**b**) Displacement measured by a dial gauge.

To conduct the lateral ballast resistance force test, the fasteners of the sleeper being measured were loosened, and the rail pad was removed. Then, the sleeper was pushed using a force loading and reading system that includes a hydraulic jack, a sensor, reacting equipment, and a digital instrument display. The lateral displacement of the rail sleeper was also measured using a dial gauge. The schematic and field test diagram are shown in Figure 3 and Figure 4.

### 2.2. Testing Results

**Figure 5:**
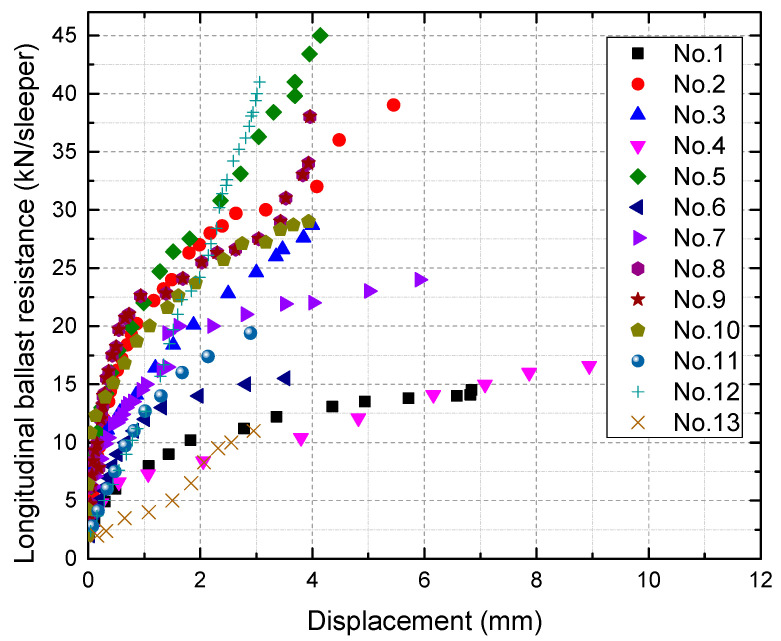
Longitudinal ballast resistance versus displacement.

**Figure 6:**
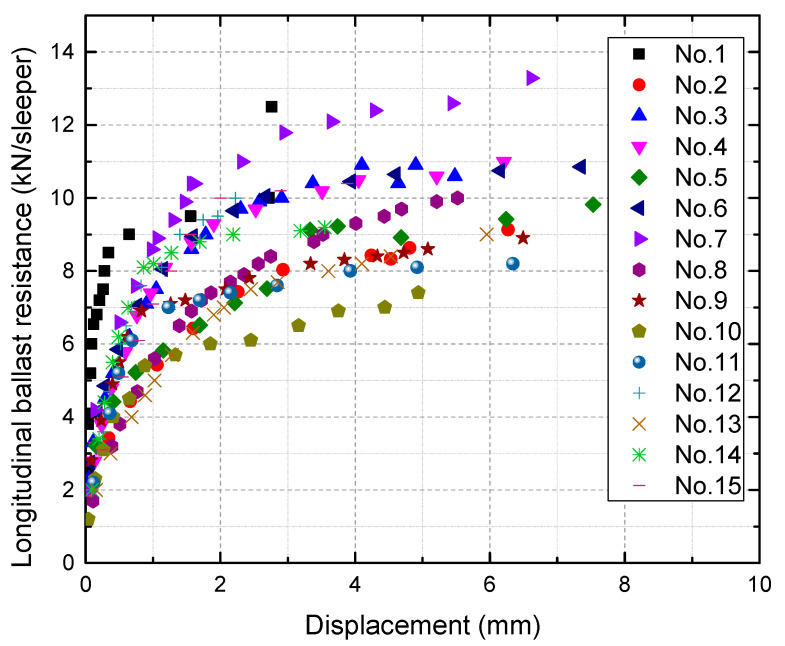
Lateral ballast resistance versus displacement.

The force-displacement relationship for longitudinal and lateral ballast resistance is presented in Figure 5 and Figure 6, respectively. As the testing is focused on small displacement (less than 8 mm), no softening or drooping behavior is observed in this test. Within the measurement range, it can be observed that the longitudinal ballast resistance displays significant variability, whereas the lateral ballast resistance exhibits relatively low variability. For both longitudinal and lateral ballast resistance, the tangent stiffness increases rapidly at first and then tends to increase more slowly.

### 2.3. SW Test for Normality of Lateral Ballast Resistance

To provide a reference for generating random lateral ballast resistance, the SW test for normality is conducted, as it is suitable for a small sample size, i.e., 15, in this case, [32]. Its general procedures are as follows:

Given *n* sample observations in ascending order $x_{\left(1\right)}⩽x_{\left(2\right)}⩽\cdots ⩽x_{\left(i\right)}⩽\cdots ⩽x_{\left(n\right)}$

Then calculate $d_{i}$, which is defined as

(2) $$d_{i}=x_{\left(n-i+1\right)}-x_{\left(i\right)},1⩽i⩽k=\{\begin{array}{c} n=2k\\ n=2k+1 \end{array}$$

The test statistic W (between 0 and 1) for normality is defined by

(3) $$W=\frac{{\left(\sum_{i=1}^{k}a_{i}d_{i}\right)}^{2}}{\sum_{i=1}^{n}{\left(x_{i}-\bar{x}\right)}^{2}}$$

where $\bar{x}$ represents the sample mean value.

$a_{i}=\left(a_{1},\cdots ,a_{n}\right)=\frac{m^{T}V^{-1}}{{\left(m^{T}V^{-1}V^{-1}m\right)}^{\frac{1}{2}}}$

$m=\left(m_{1},\cdots ,m_{n}\right)$ is composed of the expected values of the order statistics of independent and identically distributed random variables sampled from the standard normal distribution.

V is the covariance matrix of those normal order statistics.

Based on the significance level and sample size, the value of the quantile value $W_{\alpha }$ is determined. If the test statistic W is less than $W_{\alpha }$, the normality hypothesis is rejected, implying that the data does not conform to a normal distribution. Otherwise, the null hypothesis is accepted, indicating that the data follows a normal distribution.

**Figure 7:**
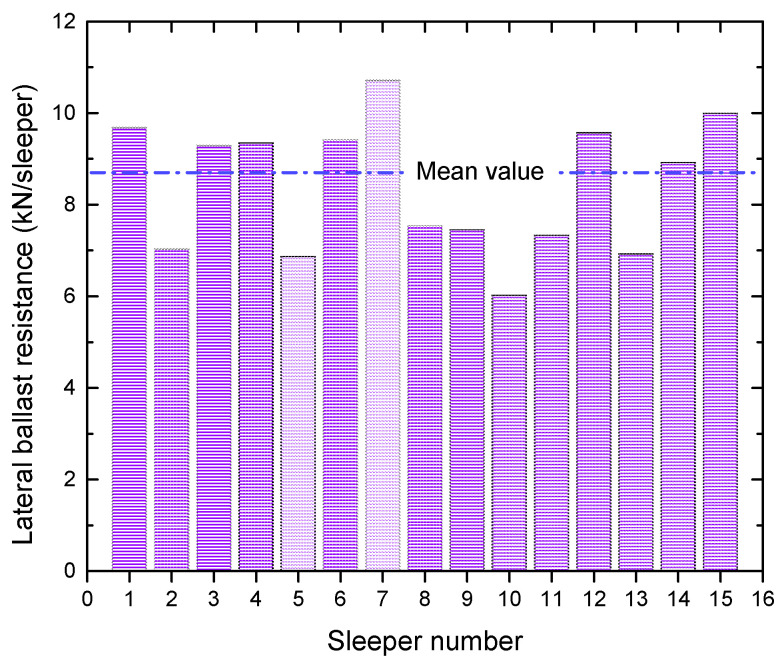
Distribution of lateral ballast resistance at 2 mm.

The SW method is then used to test the normality of lateral ballast resistance. Typically, ballast resistance is factored into calculations when the displacement of the sleeper concerning the ballast reaches a value of 2 mm [22]. However, due to the discrete nature of the data, direct acquisition of lateral ballast resistance at 2 mm from the scatter plot is not feasible. In such cases, linear interpolation is applied to calculate lateral ballast resistance at 2 mm; the results are shown in Figure 7.

The mean, variance, and standard deviation of the resulting values are 8.72, 2.49, and 1.58, respectively. The null hypothesis, H_0_, is that the overall distribution of the lateral ballast resistance samples follows a normal distribution. The test results, given a 5% level of significance, are shown in Table 1.

After calculation

$$b=\sum_{i=1}^{k}a_{i}d_{i}=5.14\;\;\;\;\sum_{i=1}^{n}{\left(x_{i}-\bar{x}\right)}^{2}=28.74\;W=\frac{b^{2}}{\sum_{i=1}^{n}{\left(x_{i}-\bar{x}\right)}^{2}}=0.919\;\;W_{\alpha }=0.881$$

$W>W_{\alpha }$, therefore accepting $H_{0}$, is indicating that the data follows a normal distribution. Knowledge of lateral ballast resistance, including its characteristics and determination, is crucial for ensuring the safety and performance of CWR. On the other hand, the statistical distribution of lateral ballast resistance can provide a reliable input parameter for further numerical investigation of this 250-m radius curve.

## 3. Numerical Study

### 3.1. Finite Element (FE) Model of CWR Track

**Figure 8:**
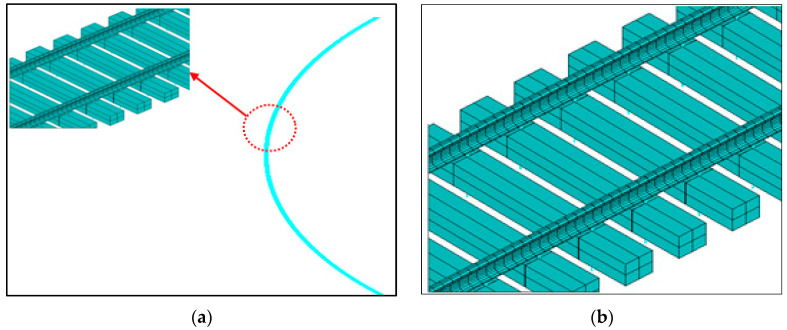
FE model of CWR track (**a**) Whole model; (**b**)FE model details of CWR track.

An FE model has been developed for a small-radius curve CWR track based on actual field conditions in Ansys. The track length considered in the model is approximately 600 m, including a certain length of tangent track, circular curve, and transition curve (see Figure 8a). The model encompasses rail, sleeper, and fastener, as well as the effect of ballast resistance, whose bottom and two ends are both fixed. The two-node beam elements based on Timoshenko’s theory are used to model the sleepers and rails. The original shape of the standard 60 kg/m rail is adopted for the section, and a simplified rectangle shape is set for the sleeper in FE modeling. Linear spring elements are used to simulate the fasteners which are placed between the rail and the sleeper. The beam elements to model rail and sleeper are assumed to be homogeneous, isotropic, and linearly elastic, and the spring element to model fasteners is linearly elastic. Based on field tests, the bilinear spring element and nonlinear spring element have been implemented to replicate the lateral ballast resistance and longitudinal ballast resistance, respectively. The input parameters for each component are reasonably adopted through relevant literature sources; refer to Table 2. The element size of the rail mesh is 0.1 m, and the element size of the sleeper mesh is from 0.58 to 1.66, as depicted in Figure 8b.

**Figure 9:**
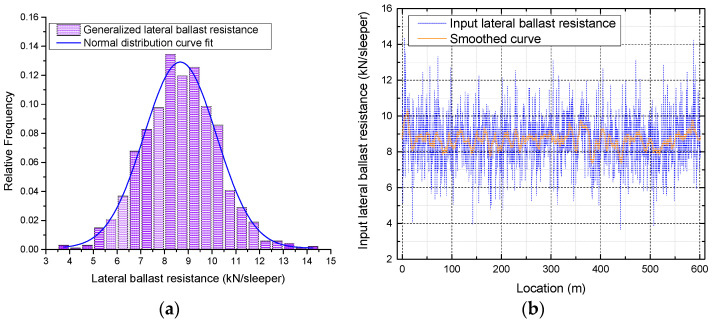
Generated normally distributed lateral ballast resistance. (**a**) Histogram of generated normally distributed lateral ballast resistance; (**b**) Randomly distributed lateral ballast resistance along the CWR track.

In the developed FE model, there are 1003 sleepers in total; hence, the lateral ballast resistance is discretized into *n* (*n* = 1003) units. To generate a set of random numbers that conform to a normal distribution with a mean of 8.72 and variance of 2.49, 1003 uniformly distributed random numbers are generated and then transformed using the Box–Muller method [33]. The distribution of generated lateral ballast resistance along the CWR track is presented in Figure 9. It is worth noting that the original input lateral ballast resistance along the CWR track undergoes smoothing techniques to indicate its trend more clearly.

The nonlinear force-displacement curve of longitudinal ballast resistance is generated by curve-fitting using the least squares method, the fitted formula is:

(4) $$r_{lo}=1.63-18.79d_{lo}+35.42d_{lo}^{3/4}$$

in which

$r_{lo}$—longitudinal ballast resistance, kN/sleeper;

$d_{lo}$—longitudinal deflection, mm.

### 3.2. Force-Deformation Characteristic Analysis

**Figure 10:**
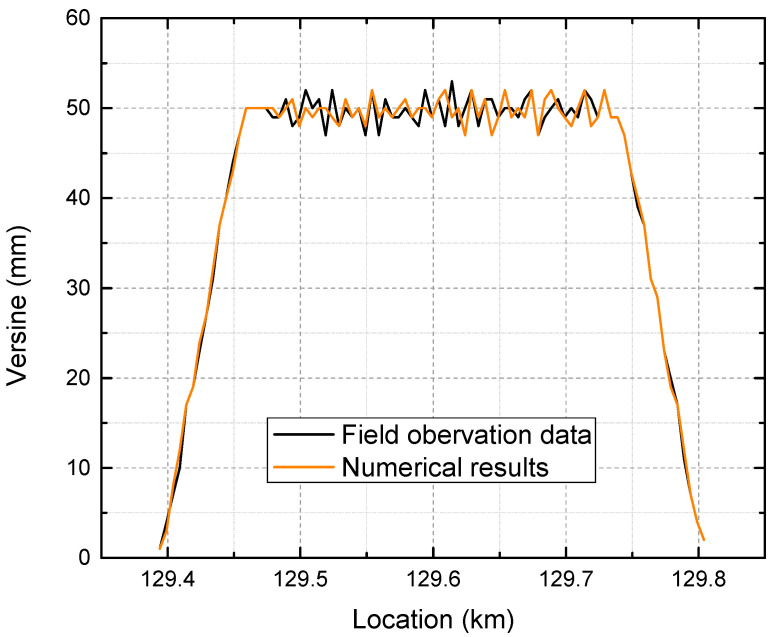
Versine distribution comparison measured by a chord length of 10 m.

Under the action of the thermal load caused by a temperature increase above the neutral temperature, the force-deformation characteristic will be analyzed. The FE model is first validated by applying a 14 °C temperature increase. The curve versine, measured using a chord length of 10 m, is compared with the field observation data, as shown in Figure 10.

**Figure 11:**
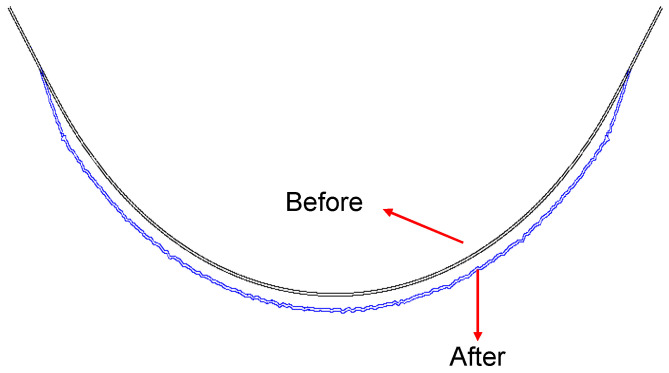
Comparison of lateral track deformation before and after 50 °C temperature increase.

**Figure 12:**
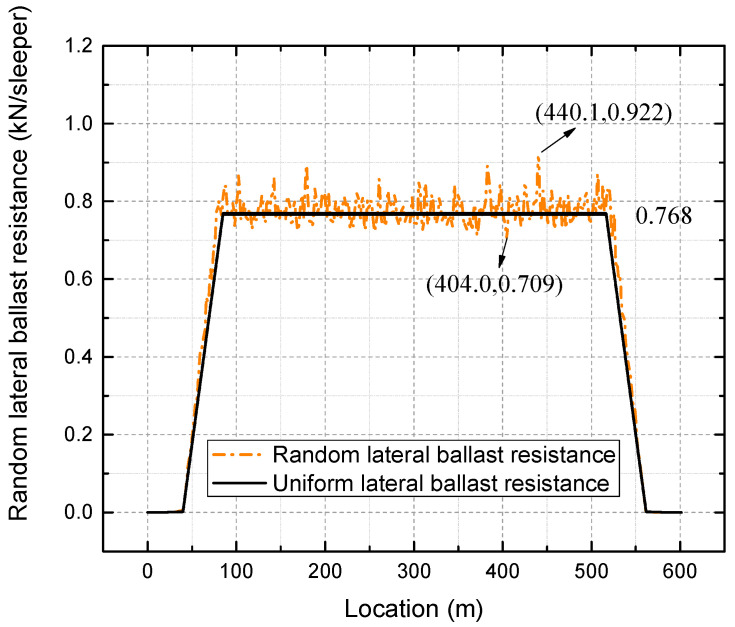
Lateral deflection after 50 °C temperature increase.

A temperature difference of 50 °C above the neutral temperature is then considered a possible loading condition at risk. Therefore, the later investigation is conducted under thermal loading of 50 °C increase. The comparison of lateral deformation before and after the temperature increase is shown in Figure 11. Figure 12 presents the comparison of lateral deflection after a 50 °C temperature increase between input uniform and random lateral ballast resistance.

**Figure 13:**
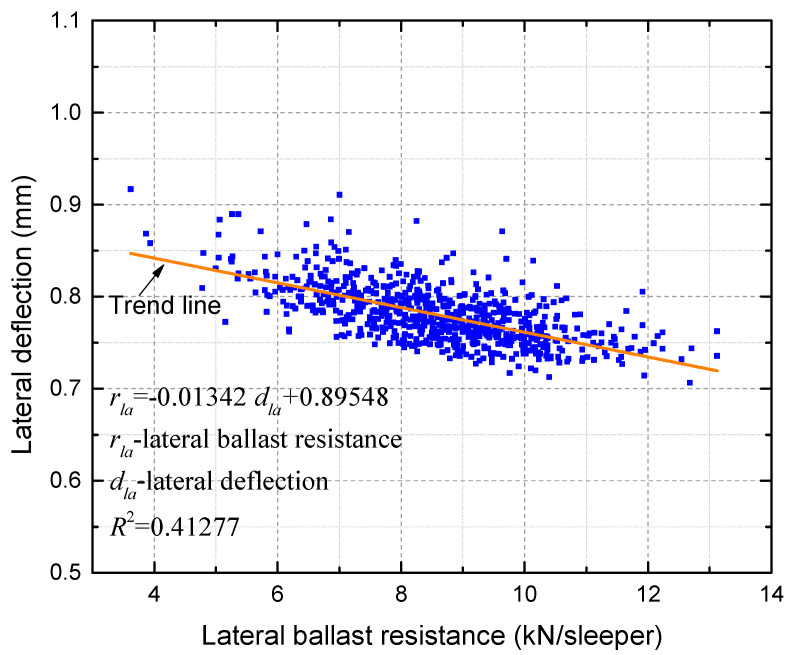
Scatterplot of lateral ballast resistance versus lateral deflection.

To explore the correlation between the smoothed lateral ballast resistance and lateral deformation, a scatter plot of lateral ballast resistance versus lateral deflection is further provided in Figure 13.

**Figure 14:**
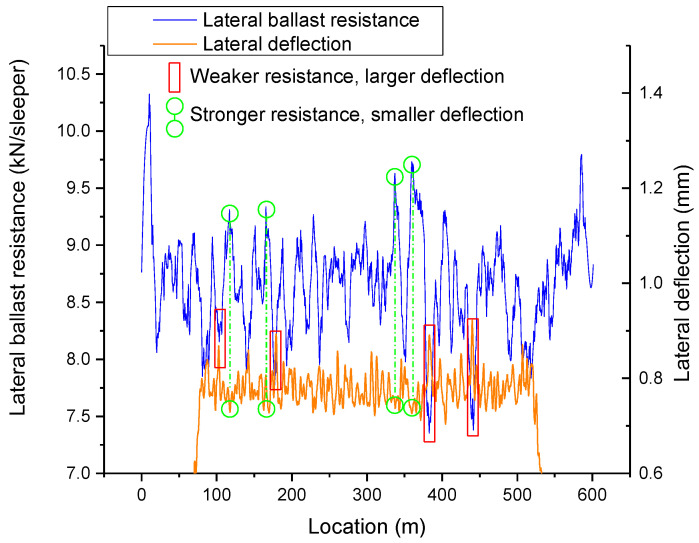
Smoothed lateral ballast resistance versus the lateral deformation along the track.

As the most concerning to maintenance is the region with maximum displacement, Figure 14 displays a contrast between the smoothed lateral ballast resistance and the lateral deformation along the CWR track.

**Figure 15:**
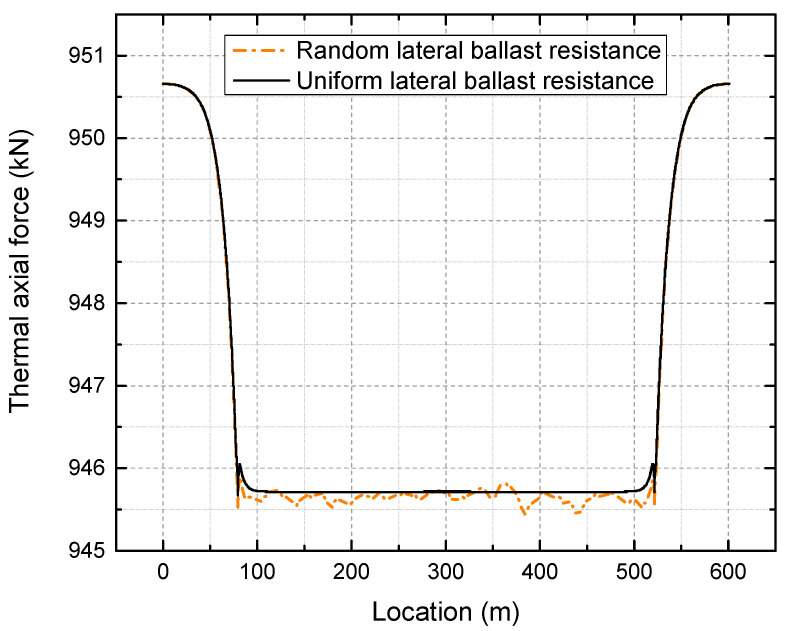
Thermal force after 50 °C temperature increase.

When temperature increases, a thermal axial force is produced inside the rail. Figure 15 illustrates the distribution of the thermal axial force at 50 °C above the neutral temperature.

**Figure 16:**
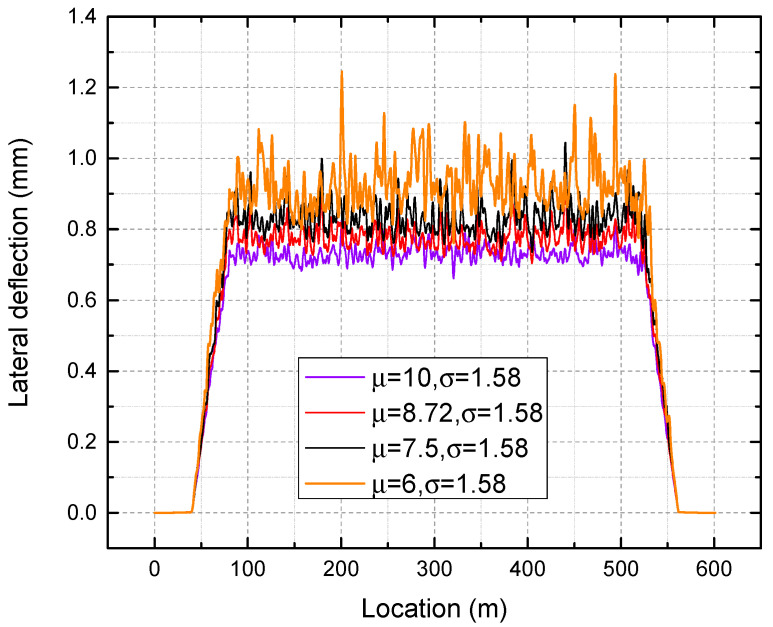
Lateral deflection under different mean values.

**Figure 17:**
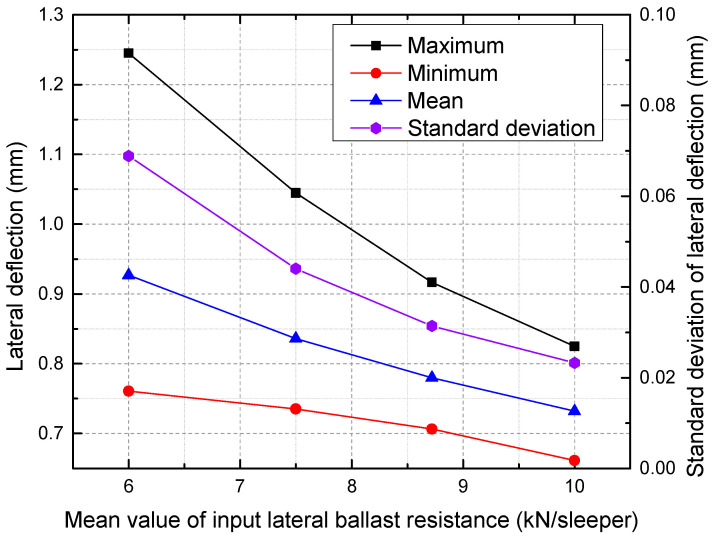
The maximum, minimum, mean, and standard deviation of lateral deflection under different mean values.

Within this framework, a parametric study is carried out to examine the effect of variations in the mean and variance of the normally distributed lateral ballast resistance. First, the standard deviation of 1.58 for the lateral ballast resistance remains constant, while the mean values are adjusted to 10, 8.72, 7.5, and 6, respectively. Figure 16 shows the lateral deflection along the CWR track under each mean value. Figure 17 presents the maximum, minimum, mean, and standard deviation of output lateral deflection under different mean values of input lateral ballast resistance.

**Figure 18:**
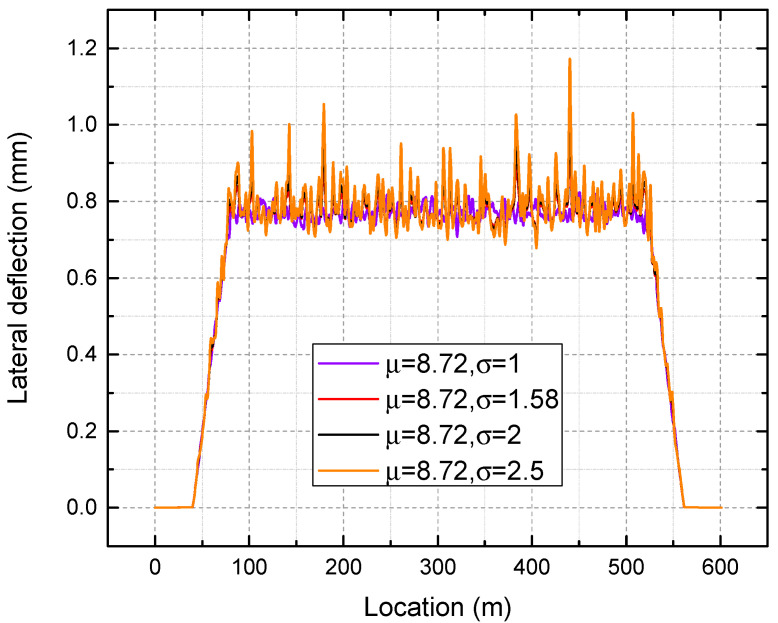
Lateral deflection under different standard deviations.

In the next step, the mean value of 8.72 is kept constant while the standard deviation of the lateral ballast resistance is varied from 1 to 2.5. Figure 18 presents lateral deflection under different standard deviations.

**Figure 19:**
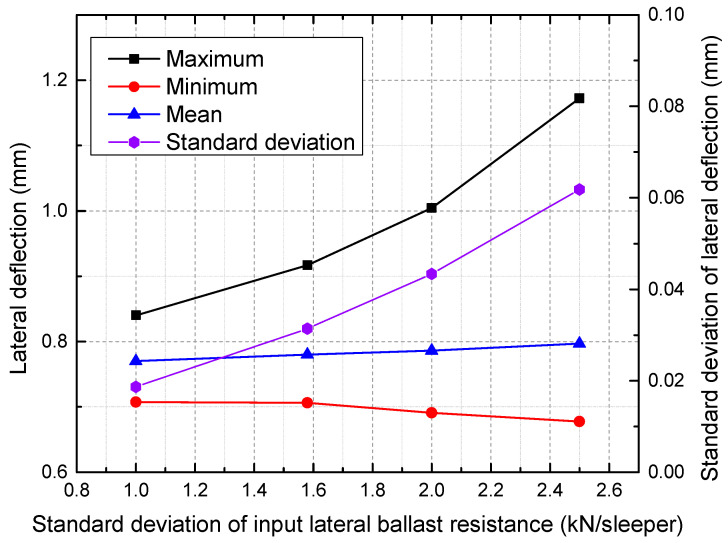
The maximum, minimum, mean, and standard deviations of lateral deflection under different standard deviations.

The maximum, minimum, mean, and standard deviations of lateral deflection are presented versus the standard deviation of input lateral ballast resistance in Figure 19.

## 4. Discussion

Figure 10 shows that the maximum and minimum values of both the numerical results and the field measurements are similar, and their randomly distributed patterns agree well. However, it should be noted that the limited testing sample and hypothetical input for lateral ballast resistance make it impossible to reproduce the exact versine distribution observed in the field.

As shown in Figure 11 and Figure 12, the lateral deformation of the track is uniform when the randomness of lateral ballast resistance is not considered. In contrast, the lateral deformation of the track becomes random instead of uniform without considering the randomness. In general, with or without the consideration of randomness, the track as a whole moves outward laterally during a temperature increase while there is almost no lateral displacement in the tangent, which is consistent with the breathing phenomenon observed in a small-radius curve. The lateral deformation in the transition curve increases from the transition start (intersection point of tangent-transition curve) to the transition end (intersection point of circular curve-transition curve). Compared with Figure 11, Figure 12 provides a more detailed comparison of the deformation between the two cases. In the absence of lateral ballast resistance randomness, the lateral displacement of the track in the circular curve remains constant at 0.768 mm. In contrast, when the randomness of the lateral ballast resistance is taken into account, the lateral track deflection in the circular curve fluctuates around 0.768 mm. The largest value of 0.922 mm is observed at location 440.1 m, while the smallest of 0.709 mm is found at location 404.0 m, representing an increase of 20.05% and a decrease of 7.68% compared to the value of 0.768 mm. Therefore, relying solely on deterministic analyses for track safety and maintenance strategies is insufficient unless worst-case scenarios for all parameters are considered.

The coefficient of determination of the trend line in Figure 13 indicates a low to moderate correlation between the lateral ballast resistance and the lateral deflection. However, it is not feasible to separate the respective performances of ballast resistance by each sleeper as they work together to resist deformation. Figure 14 demonstrates that the lateral deformation of the track is minimized in regions where the lateral ballast resistance is comparatively stronger, whereas it is higher in regions where the lateral ballast resistance is weaker. As indicated in Figure 13 and Figure 14, in the process of railway track operation and maintenance, it is of paramount importance to enhance the monitoring and maintenance of areas with weaker lateral ballast resistance.

As shown in Figure 15, without considering the random lateral ballast resistance, the actual thermal axial force inside the tangent track is almost identical to the theoretically calculated thermal axial force of 941.3 kN given in Equation (1), with an error of less than 1%. The thermal axial force in the transition curve is lower than that in the tangent track, decreasing linearly from the transition start (intersection point of tangent-transition curve) to the transition end (intersection point of circular curve-transition curve). When the random lateral ballast resistance is taken into account, the thermal axial force in the tangent track and transition curve remains the same as in the uniform case. In the circular curve, the thermal axial force is not uniform, but the difference in thermal force is less than 1 kN, which does not significantly impact the result.

It is apparent that as the mean value increases, the overall lateral deflection decreases, as shown in Figure 16. As seen in Figure 17, a reduction in the mean value of lateral ballast resistance from 10 to 6 kN/sleeper results in an increase in the maximum deflection from 0.825 mm to 1.245 mm, corresponding to a decrease of 50.9%. The same trend applies to the minimum and mean of lateral deflection. Additionally, a significant lateral ballast resistance has the ability to mitigate the variation in the lateral deflection of the track. Therefore, it can be inferred that maintaining a high-quality track alignment requires high lateral ballast resistance. A stretching of the deflection magnitude can be observed around a certain value as the standard deviation of the lateral ballast resistance is increased, as presented in Figure 18. As seen from Figure 19, an increase in the input standard deviation of lateral ballast resistance from 1.0 to 2.5 kN/sleeper results in an increase in the maximum deflection from 0.840 mm to 1.172 mm, corresponding to a decrease of 39.5%. The change of standard deviation of input lateral ballast resistance minimally affects the mean value and minimum value of lateral deflection, as observed. As the standard deviation of input lateral ballast resistance is increased from 1.0 to 2.5 kN/sleeper, the minimum deflection/mean deflection changes from 0.708/0.770 mm to 0.678/0.797 mm, corresponding to a rate of change of less than 5%.

## 5. Conclusions

This paper presents an analysis of field test data on the longitudinal and lateral ballast resistance of a 250 m-radius-curve CWR. The distribution of lateral ballast resistance is examined using the SW test, and the results indicate that it follows a normal distribution. Based on the field conditions and testing results, an FE model is developed and validated. Under thermal loading of temperature increase, after incorporating the randomness of lateral ballast resistance, the deformation of the circular curves becomes random, which more accurately reflects the actual situation and highlights the significance of incorporating the randomness of ballast resistance in the force-deformation characteristic study. A negative correlation is revealed along the CWR between input lateral ballast resistance and output lateral deflection. Regions with stronger lateral ballast resistance exhibit minimal lateral deformation of the track, while those with weaker resistance have higher lateral deformation. Consequently, improving monitoring and maintenance in areas with weaker lateral ballast resistance is of paramount importance during railway track operation and maintenance. Within this framework, parametric studies have also been conducted by varying the mean value and standard deviation of input lateral ballast resistance. High lateral ballast resistance is inferred to be required to maintain high-quality track alignment. When compared to the mean value of input lateral ballast resistance, varying its standard deviation appears to have less effect on its deformation pattern when lateral ballast resistance is maintained high.

It is worth noting that the resistance test is limited to a small displacement range, which does not include the peak and residual values. As a result, only force-deformation analysis is conducted in the numerical study. With data and distribution types of ballast resistance under large deformation conditions, buckling analysis can be further performed.

## Figures and Tables

**Table 1:** Distribution test of ballast lateral resistance at 2 mm through the SW test.

Data	$x_{\left(i\right)}$	$x_{\left(n-i+1\right)}$	$d_{i}$	$a_{i}$	$a_{i}d_{i}$	${\left(x_{i}-\bar{x}\right)}^{2}$
6.025	6.025	10.71958	4.694577	0.515	2.417707	5.701912
6.877692	6.877692	10	3.122308	0.3306	1.032235	2.356763
6.933333	6.933333	9.689655	2.756322	0.2495	0.687702	2.189022
7.036061	7.036061	9.576923	2.540862	0.1878	0.477174	1.895597
7.334783	7.334783	9.420492	2.085709	0.1353	0.282196	1.162267
7.46	7.46	9.354516	1.894516	0.088	0.166717	0.907957
7.544828	7.544828	9.296154	1.751326	0.0433	0.075832	0.753493
8.924						0.261256
9.296154						0.780195
9.354516						0.886702
9.420492						1.015307
9.576923						1.355025
9.689655						1.630187
10						2.518989
10.71958						5.32091

**Table 2:** Component properties of FE model of CWR track.

Rail	Height	176 mm
	Cross-sectional area	77.45 cm^2^
	Moment of inertia about the lateral axis	3217 cm^4^
	Moment of inertia about the vertical axis	524 cm^4^
	Elastic modulus	2.06 × 10^11^ N/m^2^
	Poisson’s ratio	0.3
Fastener	Lateral stiffness	9 × 10^6^ N/m
	Longitudinal stiffness	5 × 10^6^ N/m
	Horizontal torsional stiffness	207 N·m/rad
	Torsion moment	150N·m
Sleeper	Length	2.6 m
	Elastic modulus	3.6 × 10^10^ N/m^2^
	Sleeper spacing	0.6 m

## Data Availability

Not applicable.

## Appendix A

Given the nomenclature employed in this work, Table A1 provides a summary of the key notations adopted in this article.

**Table A1:** Nomenclature employed in this work.

Nomenclature	Description
CWR	Continuous welded rail
FE	Finite element
A	Cross-sectional area of rail
E	Elastic modulus of rail
N	Compressive force of rail
$d_{lo}$	Longitudinal deflection
$r_{lo}$	Longitudinal ballast resistance
$d_{la}$	Lateral deflection
$r_{la}$	Lateral ballast resistance
n	Number of samples
α	Thermal expansion coefficient
$x_{\left(i\right)}$	Sample observations
$\bar{x}$	Mean value of sample observations
${\left(\cdot \right)}^{T}$	Transpose of a matrix
${\left(\cdot \right)}^{-1}$	Inverse of a matrix
